# Supplementary material for: Comparative assessment of impact analysis methods applied to large commercial aircraft crash on reinforced concrete containment
Source: PLoS One. 2020 Oct 1;15(10):e0237264. doi: 10.1371/journal.pone.0237264 (PMC7529203; doi:10.1371/journal.pone.0237264)
Supplement: Attachment — Submitted filename: PONE-D-20-08713-review report.pdf [file pone.0237264.s001.pdf]

In this paper, a comparative analysis is made between force time history method and missile target interaction method for damage assessment of a large aircraft impacting reinforced concrete containment. An aircraft of Boeing 767 is greatly simplified to develop finite element model, and comparisons of impact force obtained by impact of the finite element model on the rigid wall and the Riera method are performed to verify rationality of the aircraft model. Finally, the damage of reinforced concrete containment is analyzed by two methods, including displacement， stress for concrete and steel reinforcement，and number of damaged elements. The paper is worth publishing. However, the following concerns need to be addressed in revising the manuscript:

1. In line 207~208 of this paper, “However, it has been reported by Abbas, Paul [25] that the parameter of crushing strength Pc[x(t)] has a little effect on the reaction force of rigid surface”, but in reference [25] the original sentences are that “It has been reported by others [5] that the precision required in the measurement of crushing strength is not so important. This statement may be true for some cases (as seen above) but cannot be generalised. It is clearly seen from the above discussion that the mass density and the crushing strength are equally important in the estimation of reaction time and should be measured with the same precision.” and the conclusion of reference [25] is that “Linear mass density and crushing strength significantly affect the reaction-time response, depending upon the characteristics of the aircraft (linear mass and crushing strength) and the striking velocity.” So Whether the crushing strength Pc[x(t)] is small or not for impact force of aircraft should be illustrated according to the actual situation.

2． What formula is used to calculate Pc[x(t)]? Are there some references? The crushing strength is the main factor that determines the impact time of the aircraft, please give an explanation in the paper.

3. How to obtain the velocity of the uncrushed part of the aircraft at each moment? Unfortunately, no specific process explanation is given in this paper.

4. The parameter settings of the used material model can be verified through scaling test. When the obtained parameter settings are applied in the finite element numerical calculation of the full scale model, the mesh size is usually much larger than the former. Is the influence of the mesh size on the calculation result considered in this paper? How?

5. What force time history method is applied on the containment? How is the loading area determined? Why?

6. Please explain the thickness of containment, diameter of reinforcement and reinforcement ratio.

7. How to set the bottom constraint of containment when modeling the finite element model?

8. When the force time history is loaded on the containment, the mesh size of the

containment is subdivided. How is it divided and what is the mesh size?

9. How are keywords of *MAT_NULL and *EOS_GRUNEISEN set in LS-DYNA for this paper？

10. When comparing impact displacement, concrete stress and reinforcement stress obtained with two methods, please explain the respective time of occurrence. Is there a big difference between two time of occurrence? Why?

11. In Fig.12 and Fig.15, are they resultant velocities of damaged parts? I think they may be residual velocities of uncrushed parts of aircraft.

12. In Fig.16 and Fig.20, is the selected node on steel or concrete? The displacement of scattered debris from impact area will be great without any obstruct, because the scattered debris may get an initial velocity in the impact process. It is better to give the maximum displacement of the impact area instead of scattered debris.
