# Supplementary material for: Comparative assessment of impact analysis methods applied to large commercial aircraft crash on reinforced concrete containment
Source: PLoS One. 2020 Oct 1;15(10):e0237264. doi: 10.1371/journal.pone.0237264 (PMC7529203; doi:10.1371/journal.pone.0237264)
Supplement: Attachment — Submitted filename: Reference 25.pdf [file pone.0237264.s002.pdf]

Pergamon

0045-7949(94)E0270-C

Computers & Structures Vol. 55, No. 5, pp. 809–817, 1995
Elsevier Science Ltd
Printed in Great Britain
0045-7949/95 $9.50 + 0.00

REACTION-TIME RESPONSE OF AIRCRAFT CRASH

H. Abbas,† D. K. Paul,‡ P. N. Godbole§ and **G. C. Nayak§**

†Department of Civil Engineering, Aligarh Muslim University (AMU), Aligarh 202 002, India
‡Department of Earthquake Engineering, University of Roorkee (UOR), Roorkee 247 667, India
§Department of Civil Engineering, University of Roorkee (UOR), Roorkee 247 667, India

(*Received* 12 *May* 1993)

Abstract—The safety related structures of a Nuclear Power Plant (NPP) are required to be designed for the impact of aircraft. The reaction-time response is usually obtained assuming the target to be rigid. The assumption is claimed to be conservative in the case of stiff structures. The effect of target yielding has been studied by considering an aircraft crash upon the outer containment of an NPP. It has been observed in this investigation that the assumption of a rigid target is unconservative for some of the cases.

It is usually assumed that variation in crushing strength has very little effect on the reaction from the target. However, the sensitivity analysis for reaction-time response indicates that both linear mass density and crushing strength are sensitive in affecting the reaction from the target, depending upon the characteristics of the aircraft and the striking velocity. The concept of confidence level has been investigated in relation to inclined targets also and confidence curves are obtained for aircraft.

1. INTRODUCTION

The outer containment of Nuclear Power Plants (NPP) is required to withstand the impact of crashing airplanes, aircraft debris, etc. in probabilistic considerations. Its analysis therefore requires complete information on forces exerted upon the structure by these projectiles. The pioneering work in this direction was done by Riera [1] who uncoupled the problem of aircraft crashes by considering normal impact upon a rigid target leading to the impulse which has been later used by several others [2–6] for the analysis of structures. The assumption of a rigid target disregards the inertial and stiffness properties of the target structure. Although the assumption has been claimed to be conservative by various authors [3, 7], the conclusion is based on the analysis of a rather stiff structure with insignificant local deformations. The effect of target yielding upon reaction time response has been studied in this paper by calculating the reaction simultaneously with the response of the target structure. The crashing of an aircraft upon a typical reinforced concrete (RC) outer containment of an NPP is considered for this investigation.

The subject of aircraft impact in relation to reaction-time curves is critically reviewed and drawbacks, if any, are pointed out. The probability density function for an aircraft striking an inclined surface is evaluated. The sensitivity of the reaction time response of aircraft to the variation in crushing load and linear mass density of the aircraft is studied.

In the present study, a commercial aircraft Boeing 707-320 and two military aircraft, one having low crushing strength and the other high crushing strength compared to its corresponding linear mass density, an FB-111 Jet Fighter and a Phantom F4, are considered. The variations of crushing strength and linear mass density of the three aircraft are shown in Figs 1–3.

2. LITERATURE REVIEW

The pioneering work in the area of aircraft impact on nuclear shielding structures was done by Riera [1]. In an attempt to improve upon Riera's formulation, Hornyik [5] considered energy balance as well by assuming rigid perfectly plastic behaviour of the missile material.

Rice and Bahar [8] presented an alternative derivation for calculating reaction-time history. The pitfall of their approach appears to be admitting a continuous velocity distribution throughout the control volume, in conjunction with the assumption that the thickness of the deformation zone is mathematically zero, which results in a finite jump of velocity at the missile–target interface. In fact the thickness of

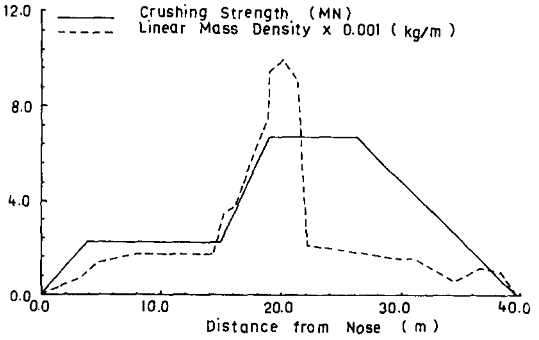

Fig. 1. Linear mass density and crushing of Boeing 707-320.

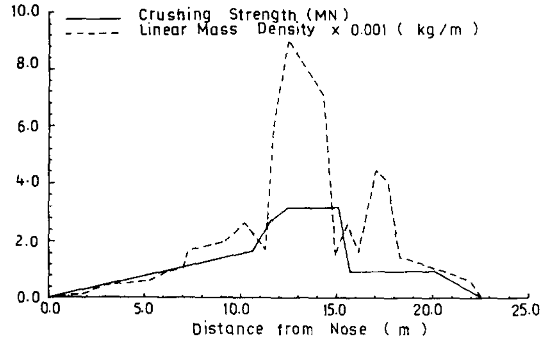

Fig. 2. Linear mass density and crushing of FB-111 jet fighter.

the deformation zone may be expected to be negligible in comparison with the length of the missile, but need not be mathematically null. In reality they found the lower limit to the reaction-time curve for Riera's model.

An attempt was made by Lange and Laue [9] to improve the material model by modelling it up to failure in a rational way. However, these refinements are questionable due to the general lack of information about the material behaviour at very high strain rates and the structural layout, etc. The hydrodynamic model used to determine the velocity of wreckage pieces does not yield conservative results [2]. An approach that involves significantly more computing effort was proposed by Drittler and Gruner [2]. This procedure was applied in [3] by the same authors to determine the reaction-time curve for a fast flying military aircraft, which was adopted as a design criterion in the Federal Republic of Germany. Bilinear elasto-plastic material behaviour and geometric linearity were assumed. The influence of the model discretisaion on the results deserves additional research.

In the most comprehensive study published to date, Wolf et al. [7] show that the reaction-time curve for a large commercial aircraft as obtained by Riera is practically indistinguishable form that obtained by modelling the aircraft with lumped masses interconnected by elasto-plastic springs.

Gardner [4] showed clearly that the effect of gravitational forces upon the reaction is negligible. He

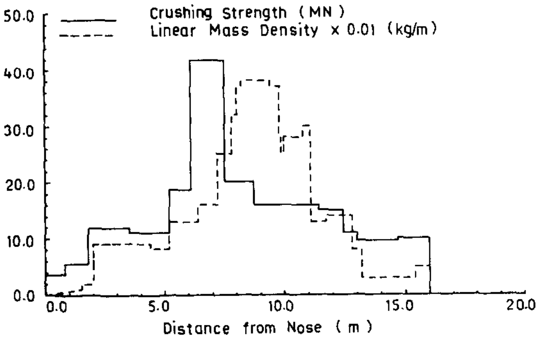

Fig. 3. Linear mass density and crushing of Phantom F4.

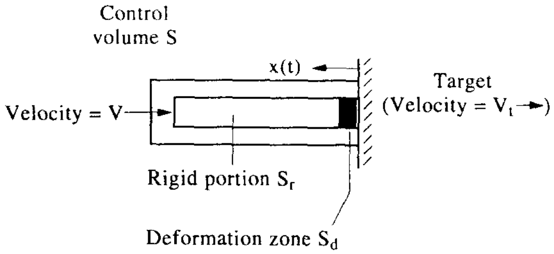

Fig. 4. Missile striking a target.

found the reaction-time curves for an oblique impact upon a vertical or horizontal target with different confidence levels. Kinsella and Jowett [10] presented a rather crude linear scaling procedure to group a range of military combat aircraft and determined the reaction response for normal impact upon a rigid target.

3. BASIC FORMULATION FOR NORMAL IMPACT

As a projectile strikes a target, a part of it close to the target gets crushed and the remaining portion of the projectile undergoes elastic deformation, which, from the point of view of deformation, may be regarded as rigid with not much error. So, consider a soft missile to consist of a thin deformation zone S_d and a rigid zone S_r within control volume S as shown in Fig. 4. The following assumptions have been made in the derivation.

(i) The aircraft is assumed as a one-dimensional model. Therefore, this model can yield only total force; it will not give variation of force on the contact area.
(ii) It is assumed that the projectile axis and its flight trajectory coincide and the impact is normal.
(iii) The aircraft is assumed to be soft. This assumption is justified because the impulse obtained for the aircraft usually lies close to the elastic projectile [4].

Assuming no torn-off wreckage pieces, no momentum crosses the boundary of the control volume.

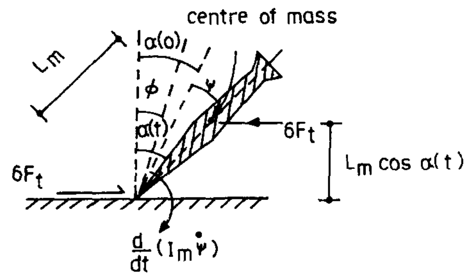

Fig. 5. Oblique strike of an aircraft.

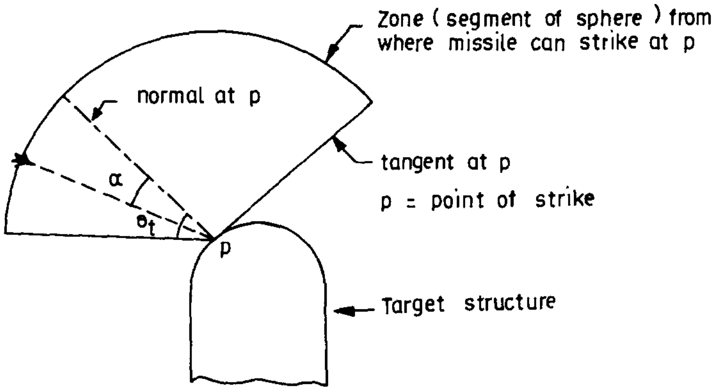

Fig. 6. Aircraft striking an inclined target.

Therefore, a particle mechanics approach can be applied to it. Application of a momentum equation to the projectile yields

$$F[x(t)] = \frac{d}{dt}(mV - m_c V_t), \quad (1)$$

where $F[x(t)]$ is the reaction from the target. If the mass entering the deformation zone remains confined without any further change, we can write

$$F[x(t)] = V\frac{dm}{dt} + m\frac{dV}{dt} - m_c\frac{dV_t}{dt}, \quad (2)$$

where V = velocity of the rigid portion of the missile at time t; V_t = velocity of the target at time t; m = mass of the rigid portion of the missile; m_c = crushed mass of the missile and mass of the target; (dm/dt) = rate at which mass enters the deformation zone = $-\mu[x(t)]V$; $\mu[x(t)]$ = mass per unit length of the missile at a distance $x(t)$ from the nose of the missile. Therefore

$$F[x(t)] = P_c[x(t)] - \mu[x(t)]V^2 - m_c\frac{dV_t}{dt}, \quad (3)$$

where $P_c[x(t)]$ is the load necessary to crush or buckle the projectile. If the target is assumed to be rigid, the reaction from the target as given by eqn (3) reduces to

$$F[x(t)] = P_c[x(t)] - \mu[x(t)]V^2. \quad (4)$$

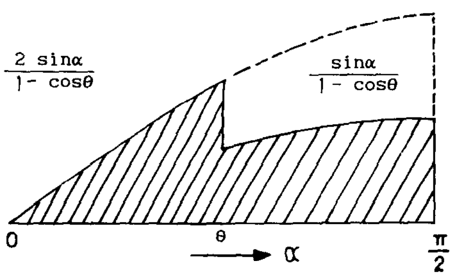

Fig. 7. Probability density function for trajectories striking a target inclined at an angle θ with horizontal.

The assumption of a rigid target leads for most cases to conservative results. However, if it happens that a flexible wall deforms in such a way that rebound motion of the wall occurs during a time interval when the load function passes through its maximum, then higher maximum values for the load are to be expected than in the case of the rigid target.

Now, let us apply the principle of conservation of energy to the control volume S at times t and $t + dt$. Loss of kinetic energy of the rigid portion in time dt is equal to the sum of work done in plastic deformation of the missile and the residual kinetic energy of the mass after crushing.

$$d(\tfrac{1}{2}mV^2) + P_c[x(t)]\,\epsilon_y\,dx - \tfrac{1}{2}dm fV^2 = 0, \quad (5)$$

where ϵ_y = yield strain; $P_c[x(t)]\epsilon_y$ = work done in plastic deformation of unit length at coordinate $x(t) = e$ (say); $\sqrt{f}$ = constant of proportionality between velocity of rigid portion V and residual velocity V' of mass after crushing. From this expression, we obtain

$$F[x(t)] = -\mu[x(t)]V^2(1+f)/2 + e. \quad (6)$$

The value of ϵ_y has been taken as unity because complete crushing of missile length dx is considered. The value of f will lie between 0 and 1. If it is assumed that the missile after crushing gets deflected by 90° (i.e. $f = 1$), this expression becomes the same as

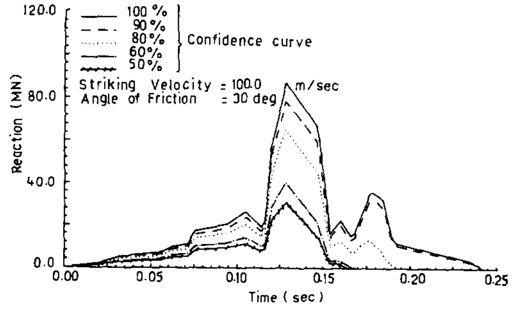

Fig. 8. FB-111 jet fighter striking a rigid target (horizontal/vertical target; normal component).

Table 1. Values of α_c for different confidence levels and inclinations of the target

Confidence level (%)	Inclination of target, θ_t (degrees)					
	0 or 90	15	30	45	60	75
50	60°0′0″	58°51′59″	55°27′35″	49°43′33″	51°19′4″	55°37′6″
90	25°50′31″	21°27′40″	19°23′11″	20°42′52″	22°19′54″	24°5′6″
95	18°11′42″	13° 3′23″	13°40′32″	14°36′26″	15°44′26″	16°58′3″

obtained in the conservation of momentum approach.

The rigid target assumption is claimed to be conservative and eqn (4) is usually followed for the determination of reaction time response. However, the claim is based on a rather stiff target. Therefore, the effect of target yielding has been investigated in a subsequent section.

4. OBLIQUE IMPACT

Although normal impact is likely to be the most unfavourable condition for the target structure, the same is not true for floor response spectra. Moreover, normal impact rarely occurs in real life and the conditional probability of normal impact is very small. Also, the orientation of the target structure may be such that the normal impact may not occur at a particular location on the target. It is for these reasons that oblique impact has also been considered in the present work.

Let α be the inclination of the aircraft to the normal and ϕ be the angle of friction between the target and the missile, which is assumed to be reliably estimated. If $\alpha < \phi$, the impact point will be stationary and the normal F_n and tangential F_t reaction can be found from the relations

$$F_n = F[x(t)]\cos\alpha \text{ and } F_t = F[x(t)]\sin\alpha. \quad (7)$$

On the other hand, when $\alpha > \phi$, an unbalanced tangential force δF_t appears at the target–missile interface and causes the contact point to move and rotates the missile, δF_t being given by

$$\delta F_t = F[x(t)](\sin\phi - \sin\alpha). \quad (8)$$

Since α changes with time, for this particular case α has been replaced by $\alpha(t)$. The application of classical dynamics for the rotation of the missile (Fig. 5) leads to

$$I_m\frac{d\theta^2}{dt^2} + \frac{dI_m}{dt}\frac{d\theta}{dt} = F[x(t)]L_m\cos\alpha(t) \times (\sin\phi - \sin\alpha(t)), \quad (9)$$

where θ is the change in the angle of inclination at time t such that $\alpha(t) = \alpha(0) + \theta$; L_m is the distance of the centre of mass of the rigid portion of the missile from the instantaneous point of impact and I_m is the mass moment of inertia of the uncrushed portion of the aircraft with respect to the centre of mass. The expression (9) has been used for calculating the change in the angle of inclination θ.

5. CONFIDENCE LEVEL FOR IMPACT REACTION

Considering the position of a flying aircraft as a random variable in three-dimensional space, the probability density function $p(\beta)$ for an aircraft crash striking the structure may be defined by

$$p(\beta) = \frac{N'(\beta)}{N'(\pi/2)} \quad (10)$$

$N(\alpha)$ = number of trajectories striking the target between 0 and α

$$N'(\alpha) = \frac{dN(\alpha)}{d\alpha}. \quad (11)$$

Therefore, the probability P of an aircraft striking the structure at an angle less than or equal to β is

$$P(\beta) = \frac{N(\beta)}{N(\pi/2)}, \quad (12)$$

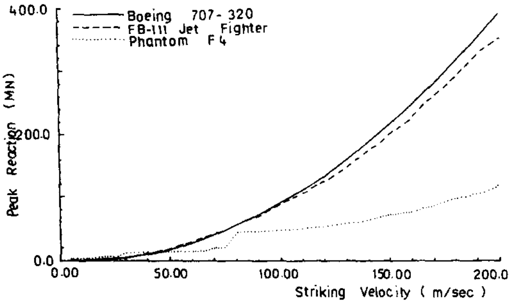

Fig. 9. Striking velocity vs peak reaction.

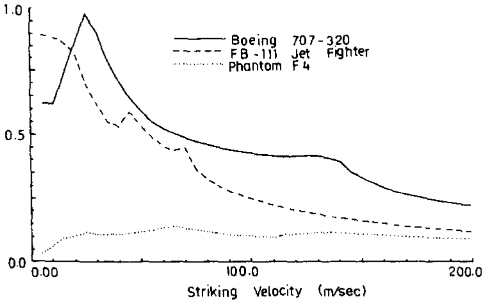

Fig. 10. Striking velocity vs impact duration.

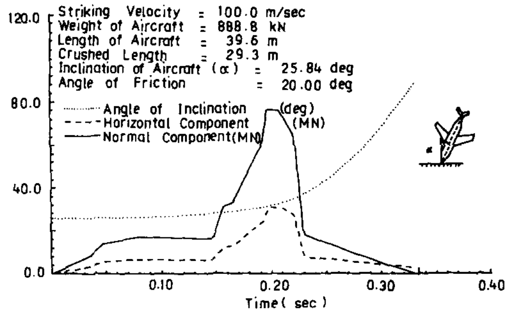

Fig. 11. Boeing 707-320 striking a rigid target (horizontal/vertical target; 90% confidence curves).

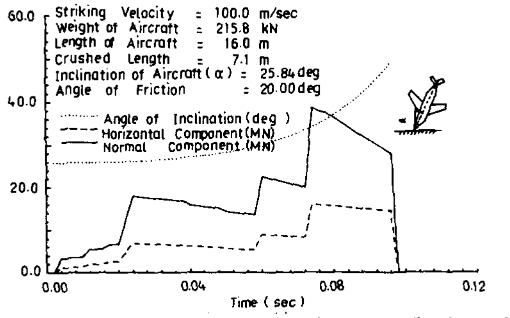

Fig. 13. Phantom F4 striking a rigid target (horizontal/vertical target; 90% confidence curves).

where

$$N(\beta) = \int_0^{\beta} N'(\alpha)\,d\alpha.$$

It is believed that an isotropic trajectory distribution is most relevant to the air crash problem. The consideration of a hemisphere by Reira [11] and Gardner [4] is true only for aircraft striking a horizontal or vertical surface. However, we shall consider a segment of a sphere for an aircraft striking an inclined target (Fig. 6). The probability density function for such a case has been evaluated as explained below.

The area of a hypothetical segment of a sphere from which the trajectories can come is between the angles β and $\beta + d\beta$ to the target whose normal is inclined at an angle θ_t with the horizontal. The probability density function for an isotropic trajectory distribution upon a structure is obtained as (Fig. 7)

$$N'(\alpha) = \frac{2\sin\alpha}{2-\cos\theta_t} \quad \text{for } \alpha \leqslant \theta_t \qquad (13)$$

$$= \frac{\sin\alpha}{2-\cos\theta_t} \quad \text{for } \alpha > \theta_t. \qquad (14)$$

If c% trajectories have an angle greater than an angle α_c then the normal component of the reaction curve corresponding to α_c will envelope c% of the normal reaction curves (see Fig. 8). The reaction curve corresponding to the angle α_c will have c% confidence.

When $\alpha_c < \theta_t$

$$\frac{c}{100} = \int_{\alpha_c}^{\pi/2} N'(\alpha)\,d\alpha$$

$$= \int_{\alpha_c}^{\theta_t} \frac{2\sin\alpha}{2-\cos\theta_t}\,d\alpha + \int_{\theta_t}^{\pi/2} \frac{\sin\alpha}{2-\cos\theta_t}\,d\alpha$$

$$= \frac{2\cos\alpha_c - \cos\theta_t}{2-\cos\theta_t} \qquad (15)$$

whereas, for $\alpha_c \geqslant \theta_t$

$$\frac{c}{100} = \int_{\alpha_c}^{\pi/2} \frac{\sin\alpha}{2-\cos\theta_t}\,d\alpha = \frac{\cos\alpha_c}{2-\cos\theta_t}. \qquad (16)$$

The values of α_c for different confidence levels and inclinations of the target have been worked out and are given in Table 1.

The most severe normal impact for c% confidence occurs when $\theta_t = \alpha_c$ and it is given by the relation

$$(\alpha_c)_{\min} = \frac{2c}{100+c}. \qquad (17)$$

Equation (17) gives the values of $(\alpha_c)_{\min}$ for 50, 90 and 95% confidence levels as 48°11′23″, 18°40′18″ and 13°0′10″, respectively. For a given value of α_c, c will be maximum when $\theta_t = 90°$, whereas, for a given value of θ_t, c will be maximum when $\alpha_c = 0°$.

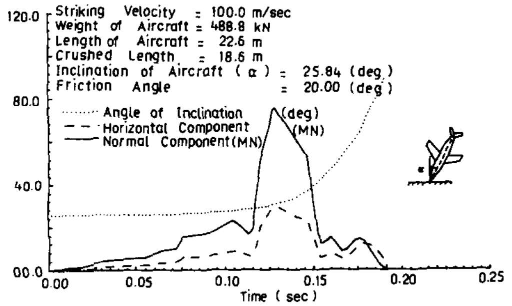

Fig. 12. FB-111 fighter striking a rigid target (horizontal/vertical target; 90% confidence curves).

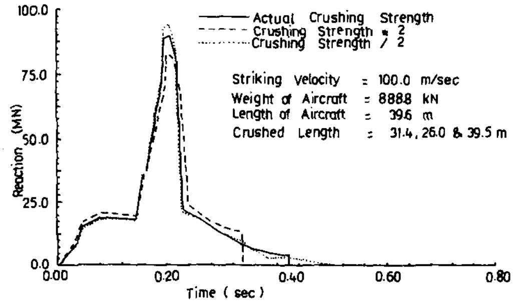

Fig. 14. Normal impact of Boeing 707-320 upon a rigid target (effect of variation in crushing strength).

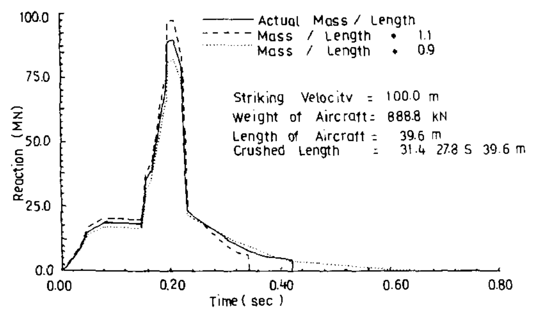

Fig. 15. Normal impact of FB-111 fighter upon a rigid target (effect of variation in crushing strength).

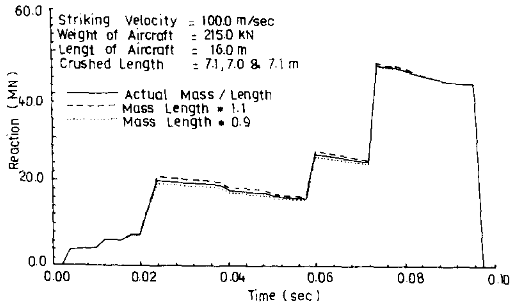

Fig. 17. Normal impact of Boeing 707-320 upon a rigid target (effect of variation in mass/length).

6. NUMERICAL SOLUTION

Numerical finite difference approach has been employed for determining the reaction from the target using the above described equations. The impact process continues until any one of the following conditions are met:

(i) velocity of aircraft diminishes to zero;
(ii) angle of inclination of aircraft from normal to the target surface reaches 90° limit;
(iii) missile gets completely crushed.

The variations of peak reaction and impact duration for the normal impact of three aircraft upon an unyielding target are shown in Figs 9 and 10. It is seen from these figures that the peak reaction increases with increase in velocity. However, the rate of increase for the Boeing and FB-111 aircraft is nearly the same, whereas it is much slower in case of the Phantom F4. Impact duration of the Boeing and FB-111 usually decreases but it is nearly constant for the Phantom F4. This behaviour for the Phantom F4 may be attributed to the sharp variations in crushing strength and linear mass density.

The horizontal and vertical components of the reaction-time curves for the three aircraft under study striking a horizontal or vertical target at 100 m/sec velocity for the 90% confidence level are shown in Figs 11–13. The change in inclination due to the rotation of the aircraft is also shown in these figures. As an aircraft strikes at an inclination α to the target, its angle of inclination remains more or less constant until such time when the missile velocity reduces significantly. Beyond this, the inclination increases at a faster rate which results in a faster rate of reduction of the reaction for all three cases.

7. INFLUENCE OF VARIATION IN BUCKLING LOAD AND LINEAR MASS DENSITY

The sensitivity of the reaction time responses of an aircraft to the variation in crushing load and linear mass density of the aircraft has been studied. The crushing strength is magnified and reduced by a factor of two and linear mass density is varied by 10%. The response of this variation for the three aircraft under study is shown in Figs 14–19. The contribution of buckling load towards total reaction can be seen in Figs 20–25 for the three aircraft under study for two velocities, 50 and 100 m/sec. It is seen from these plots that for stiffer aircraft the contribution of crushing strength towards reaction is greater, which further increases with decrease in velocity.

The influence of the buckling load for 50% variation and the linear mass density for 10% variation are also shown in Table 2.

It has been reported by others [5] that the precision required in the measurement of crushing strength is not so important. This statement may be true for some cases (as seen above) but cannot be generalised. It is clearly seen from the above discussion that the mass density and the crushing strength are equally

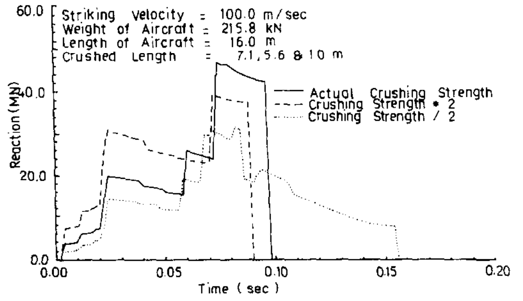

Fig. 16. Normal impact of Phantom F4 upon a rigid target (effect of variation in crushing strength).

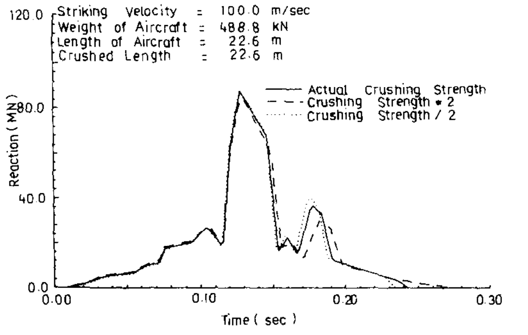

Fig. 18. Normal impact of FB-111 fighter upon a rigid target (effect of variation in mass/length).

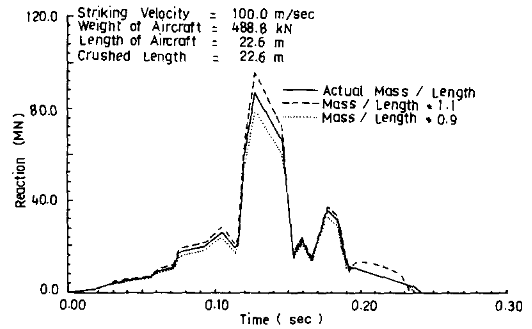

Fig. 19. Normal impact of Phantom F4 upon a rigid target (effect of variation in mass/length).

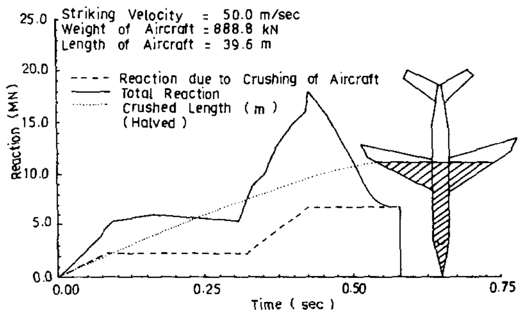

Fig. 20. Boeing 707-320 striking a rigid target (normal impact).

important in the estimation of reaction time and should be measured with the same precision.

8. EFFECT OF TARGET YIELDING

The effect of target yielding is studied by simultaneously calculating the reaction following relation (3). Outer containment of a typical NPP (Fig. 26), analysed by Rebora *et al.* [12] for the horizontal impact of the Boeing 707-320 at 230 mph, has been considered in the present study.

The geometry of the containment shell, reinforcement details and corresponding mesh discretisation are shown in Fig. 26. The cylindrical shell and the spherical top dome are of a constant thickness of 1.2 m. The shell is doubly reinforced with equal reinforcement on the inner and outer faces consisting of 40 mm diameter bars spaced at a c/c distance of 80 mm (1.1%) placed circumferentially and meridionally in the cylindrical shell as well as in the spherical top dome. The material properties used in the analysis are taken from [12].

The shell is discretised into 52, 20-noded isoparametric brick elements with a total of 342 nodes leading to 1100 degrees of freedom of the structure. The horizontal impact of a Boeing 707-320 with a striking velocity of 102.82 m/sec (230 mph) on the RC containment is analysed. The area of impact is assumed to be 28 m^2.

The reaction-time responses for the rigid target and the yielding target for the horizontal crashing of the three aircraft are plotted in Fig. 27. It is seen from these plots that reaction-time response is almost unaffected initially. However, when the peak reaction is reached, the response of the target becomes more pronounced, thereby affecting the reaction-time

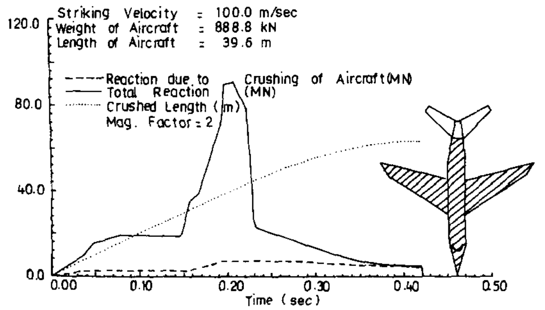

Fig. 21. Boeing 707-320 striking a rigid target (normal impact).

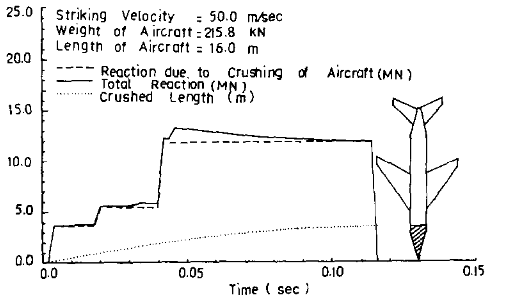

Fig. 22. FB-111 fighter striking a rigid target (normal impact).

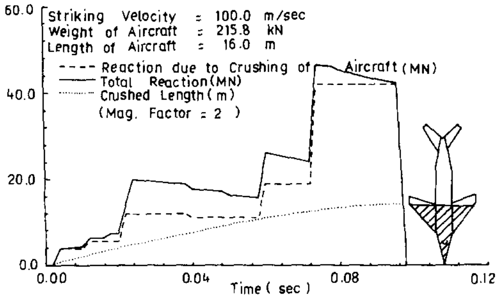

Fig. 23. FB-111 fighter striking a rigid target (normal impact).

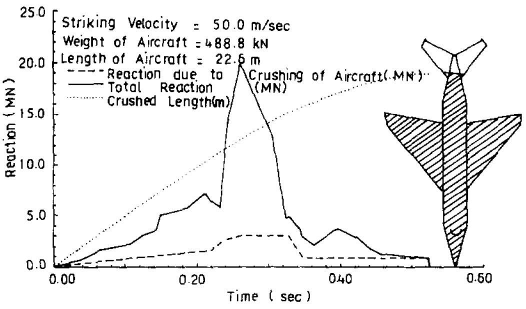

Fig. 24. Phantom F4 striking a rigid target (normal impact).

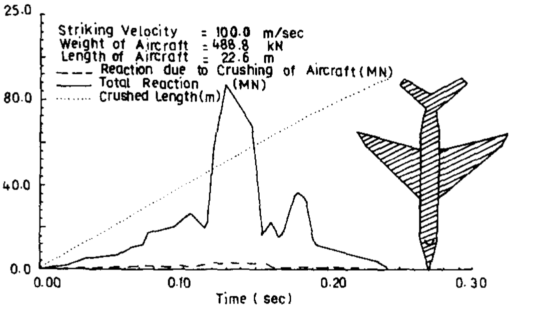

Fig. 25. Phantom F4 striking a rigid target (normal impact).

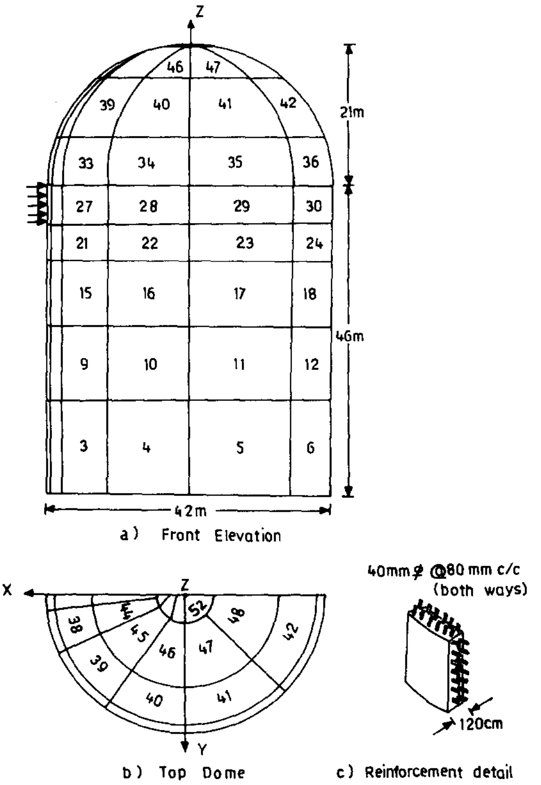

Fig. 26. Dimensions, discretisation and impact location.

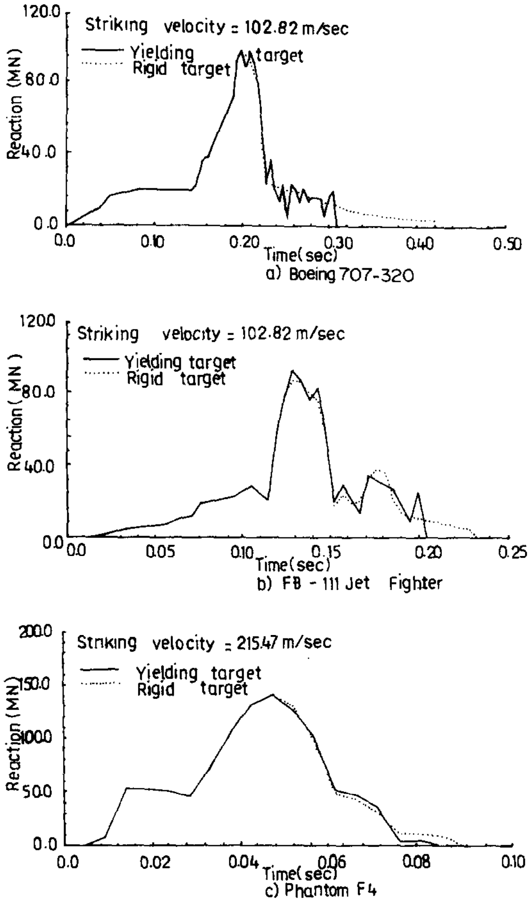

Fig. 27. Effect of yielding of target upon reaction time for the three aircraft.

response. When the decline in the reaction is sharp, the target response will still be high but the velocity of the missile is considerably reduced, as a result of which the effect on the reaction becomes more pronounced, as in the case of the Boeing 707-320. In the case of the Phantom F4, the decline in the reaction is not so sharp, therefore the variation is not so noticeable. It is further noticed that the reaction-time response obtained on the basis of a rigid target is not even conservative. In the case of a slender target, the effect of target response upon the reaction may be even more pronounced. It is therefore undesirable to use the reaction-time response obtained on the basis of a rigid target concept as is the usual practice.

Table 2. Influence of crushing strength and linear mass density (striking velocity = 100 m/sec)

Particulars	Boeing 707	FB-111 jet fighter	Phantom F4
1. ±50% *variation in crushing strength*			
(a) Change in peak load	−4.2%	−3.1%	+36.6%
	+4.8%	+0.9%	−31.7%
(b) Change in impact duration	−14.7%	+5.8%	−6.1%
	+32.7%	−3.3%	+59.2%
2. ±10% *variation in linear mass density*			
(a) Change in peak load	+8.3%	+9.5%	+0.9%
	−8.7%	−9.5%	−1.0%
(b) Change in impact duration	−19.0%	−3.3%	0.0%
	+50.2%	−1.7%	0.0%

9. CONCLUSIONS

It is noticed that the reaction-time response obtained on the basis of a rigid target is not only unrealistic but also unconservative. In the case of a slender target, the effect of target response upon the reaction will be more pronounced. The effect of target yielding is dependent upon both the characteristics of the target and those of the missile. It is therefore undesirable to use the reaction-time response obtained on the basis of a rigid target concept as is the usual practice.

The concept of confidence level has been investigated in relation to inclined targets also and confidence curves have been obtained for the aircraft. Linear mass density and crushing strength significantly affect the reaction-time response, depending upon the characteristics of the aircraft (linear mass and crushing strength) and the striking velocity.

Acknowledgements—The first author wishes to thank his parent institute, Aligarh Muslim University, for granting leave to pursue a Ph.D. under the Quality Improvement Program (QIP) scheme of the University Grants Commission (UGC), India.

REFERENCES

1. J. D. Riera, On the stress analysis of structures subjected to aircraft impact forces. *Nucl. Engng Des.* **8**, 415–426 (1968).
2. K. Drittler and P. Gruner, Calculation of the total force acting upon a rigid wall by projectiles. *Nucl. Engng Des.* **37**, 231–244 (1976).
3. K. Drittler, P. Gruner and J. Krivy, Calculation of forces arising from impacting projectiles upon yielding structures. 4th SMiRT, San Francisco, U.S.A., Paper No. J7/4 (1977).
4. J. W. Gardner, Calculation of the forces acting upon a rigid structure from an aircraft impact. *Int. J. Impact Engng* **2**(4), 345–356 (1984).
5. K. Hornyik, Analytic modeling of the impact of soft missiles on protective walls. 4th SMiRT, San Francisco, U.S.A., Paper No. J7/3 (1977).
6. R. P. Kennedy, A review of procedures for the analysis and design of concrete structures to resist missile impact effects. *Nucl. Engng Des.* **37**, 183–203 (1976).
7. J. P. Wolf, K. M. Bucher and P. E. Skrikerud, Response of equipment subjected to aircraft impact. *Nucl. Engng Des.* **47**, 169–193 (1978).
8. J. S. Rice and L. Y. Bahar, Reaction-time relationship and structural design of reinforced concrete slabs and shells for aircraft impact. 3rd SMiRT, London, U.K. Paper No. J5/3 (1973).
9. L. Lange and H. Laue, Dynamische Belastung eines Reaktorgebaudes infolge Flugzeugabsturz. 2nd SMiRT, Berlin, Paper No. I3/2 (1973).
10. K. Kinsella and J. Jowett, The dynamic load arising from a crashing military combat aircraft. Proceedings of First International Conference on Structure under Shock and Impact (Edited by P. S. Bulson), Cambridge, MA, pp. 277–288. Elsevier, Amsterdam (1989).
11. J. D. Riera, A critical reappraisal of nuclear power plant safety against accidental aircraft impact. *Nucl. Engng Des.* **57**, 193–206 (1980).
12. B. Rebora, Th. Zimmermann and J. P. Wolf, Dynamic rupture analysis of reinforced concrete shells. *Nucl. Engng Des.* **37**, 269–297 (1976).
