# Supplementary material for: Comparative assessment of impact analysis methods applied to large commercial aircraft crash on reinforced concrete containment
Source: PLoS One. 2020 Oct 1;15(10):e0237264. doi: 10.1371/journal.pone.0237264 (PMC7529203; doi:10.1371/journal.pone.0237264)
Supplement: Attachment — Submitted filename: Response to reviewers_Final.docx [file pone.0237264.s003.docx]

**Authors’ Response to Reviewer’s Comments**

**PONE-D-20-08713**

**Title:  Comparative assessment of impact analysis methods applied to large commercial aircraft crash on reinforced concrete containment**

**Muhammad Sadiq, Rao Arsalan Khushnood, Muhammad Ilyas, Wasim Khaliq, Shaukat Ali Khan, and Pan Rong**

The authors appreciate the constructive suggestions by the reviewers. We agree with most of the reviewer’s suggestions and technical comments. The manuscript has been revised to incorporate suggestions by the reviewer. In the revised paper, the changes (additions) are made in track change mode. The following explains the authors’ response to the reviewers’ comments.

| **Sr. No** | **Reviewer’s comment** | **Authors’ response** |
| --- | --- | --- |
| 1. | In line 207~208 of this paper, “However, it has been reported by Abbas, Paul [25] that the parameter of crushing strength Pc[x(t)] has a little effect on the reaction force of rigid surface”, but in reference [25] the original sentences are that “It has been reported by others [5] that the precision required in the measurement of crushing strength is not so important. This statement may be true for some cases (as seen above) but cannot be generalized. It is clearly seen from the above discussion that the mass density and the crushing strength are equally important in the estimation of reaction time and should be measured with the same precision.” and the conclusion of reference [25] is that “Linear mass density and crushing strength significantly affect the reaction-time response, depending upon the characteristics of the aircraft (linear mass and crushing strength) and the striking velocity.” So, Whether the crushing strength Pc[x(t)] is small or not for impact force of aircraft should be illustrated according to the actual situation. | The authors do agree with the prestigious reviewer. The influence of crushing strength Pc[x(t)] on impact forces and impulses of aircraft are evaluated for current study and following highlighted text along with curves in Figs. 5 and 6 are added in lines 221-241 of revised manuscript, as recommended please.  In current study, the influence of the crushing strength on the impact forces and impulses are evaluated by using the Riera equation [1]. The actual crushing strength of Boeing 707-320, as adopted in reference [2] with four different percentages, is considered in calculation of impact forces and impulses. The calculations are performed for the two impact velocities of 100 m/s and 150 m/s as show in Figs. 5 and 6. For both cases, the impact force and impulse time history curves are quite similar. At impact velocity of 100 m/s shown in Fig. 5, maximum variation of 1.16 % in impact forces and 5.50 % in impulses are observed due to four varying crushing strengths. As shown in Fig. 6, the variation in impact forces and impulses are further reduced to 0.40 % and 2 % at higher impact velocity of 150 m/s. It is observed that these small variations in impact forces and impulses due to varying crushing strengths would have insignificant influence. Mainly, mass and velocity of the aircraft dominate/control the force-time history and are referred as the controlling cases. Since the crushing strength of the Boeing 767 aircraft model is not available in open literature, therefore, it is acceptable approach to adopt the crushing strength of available Boeing 707-320 from reference [2]. T. Zhang et al [3] also utilized the crushing strength of Boeing 707-320 in impact simulation of A320 aircraft after determining its influence on impact forces and impulses by similar approach.  Moreover, Jorma Arros and Nikolay Doumbalski (authors of reference 5 of manuscript) also mentioned that Crushing force can be quite approximate and insensitive to force time history (a relevant slide is attached at Annexure-I below). The same conclusion is drawn in current study as mentioned above, please. |
| 2. | What formula is used to calculate Pc[x(t)]? Are there some references? The crushing strength is the main factor that determines the impact time of the aircraft, please give an explanation in the paper. | As mentioned in line 236-237, available crushing strength of Boeing 707-320 is taken from reference [2]. This is done after evaluating the influence of crushing strength on impact forces and impulses as mentioned in response of comment 1, please.  Regarding the impact time/duration lines 274-276 state that “Impact duration of 150 m/s impact velocity case is shorter, but impact forces and impulses are more due to higher velocity of aircraft, i.e. $\upsilon^{2}(t)$in equations (1) and (2)”.  The below highlighted text is also added in lines 276-280 of revised manuscript to further address the concern as suggested, please.  Similarly, Liu et al [4] reported that with increasing impact velocity, the duration of the total impact force reduces but the peak value of the total impact force increases significantly. This indicates that velocity is the main factor determining the impact duration as the length of the aircraft is same during both impact velocities i.e. 100 m/s and 150 m/s. |
| 3. | How to obtain the velocity of the uncrushed part of the aircraft at each moment? Unfortunately, no specific process explanation is given in this paper. | As highlighted by the kind reviewer, the related process involved in the estimation of residual velocities of uncrushed parts of aircraft is explained in lines 307-315 of revised manuscript, please:  The residual velocities of uncrushed aircraft parts are obtained by using *DATABASE_BINARY_D3THDT option available in LS-DYNA [5]. At defined output time intervals (e.g., DT=0.03 sec in current analysis), velocity time history file is genaerated for all defined parts of aircraft. The residual velocity time histories extracted from this file are plotted for individual parts (such as fuselage, wings and engine) as shown in Figs. 14 and 17. According to NEI 07-13 [6], the term “residual velocity” is the exit velocity of missile/aircraft that has an initial velocity greater than the perforation velocity. In experimental studies such as performed by Jun Mizuno et al [7], the residual velocity times histories of uncrushed aircraft parts are obtained by means of on-board accelerometers and high-speed video cameras. |
| 4. | The parameter settings of the used material model can be verified through scaling test. When the obtained parameter settings are applied in the finite element numerical calculation of the full-scale model, the mesh size is usually much larger than the former. Is the influence of the mesh size on the calculation result considered in this paper? How? | Reference lines 179-186 of manuscript, it is mentioned that the main objective of simulation analysis performed by author in 2014 [8] was the validation of constitutive models with test results for full scale impact analysis against large commercial aircraft. Based on that study, two material models, Winfrith concrete model (*MAT_084) and Continuous Surface Cap Model (CSCM) model (*MAT_159) were used. Since the results of Winfrith concrete model (*MAT_084) corresponded well with the experimental results [8], hence the same model is selected for concrete of outer containment building in this study with the input parameters shown in Table 2 of manuscript. For aircraft parts, lines 155-157 of manuscript also mentioned that Material model, *MAT_PLASTIC_KINEMATIC (*MAT_003) available in LS-DYNA [5] is used, after validation of constitutive models with test results by author [8] and the input parameters are mentioned in Table 1.  Now, the mesh size in current full scale impact analysis is larger than adopted in simulation analysis of 1/7.5 scale model tests [8] and its effects are checked by performing various analysis runs of defined aircraft model on rigid wall. The time histories of the impact forces are compared with Riera curves. Most comparable force and impulse time histories were obtained once the mesh size of 400 mm for fuselage & wings and 200 mm for engines were selected in aircraft modelling. Further refinement of mesh sizes did not lead to significant improvement to the solution. Therefore, mesh sizes of 400 mm for fuselage & wings and 200 mm for engines are chosen in the full-scale simulation analysis as shown in Fig. 2(b). The said highlighted text is added in lines 146-154 of revised manuscript as suggested, please.  Moreover, the results of rigid wall impact analysis in section 4 of manuscript show that FE model of Boeing 767 has almost similar characteristic to actual aircraft and meets the recommendation set forth in NEI 07-13 [6]. These results corresponded well with Riera method, which also verifies the accuracy of selected mesh sizes in full scale simulated analysis. |
| 5. | What force time history method is applied on the containment? How is the loading area? determined? Why? | With reference to lines 245-246 and 347-348 of manuscript, it is mentioned that the Riera’s force time history curves shown in Fig. 11 of manuscript for α=1, corresponding to two speeds of 100 m/s and 150 m/s, are applied to a zone of outer containment building as highlighted in Fig.7.  As illustrated in Fig. 1. below, the loading area (also called impact area) is determined by using the front view dimensions of Boeing 767. The rectangular loading area (A) corresponds to where the wings and engine strike and the approximate circular area (B) is impacted by the fuselage. The forces were applied as pressure loads in ANSYS/LS-DYNA.    **A**  **B**  Figure 1: Illustration of loading area on containment building determined from dimensions of Boeing 767.  J. Arros, N. Doumbalski [9] also used the similar approach for simulation of Riera force time history, please see the below Fig. 2.    Figure 2: Loading area/impact area for Riera force time history method as adopted by J.Arros et al [9].  In current paper, the said approach is followed in order to simulate the Riera force time history for comparison of results with missile target interaction method. |
| 6. | Please explain the thickness of containment, diameter of reinforcement and reinforcement ratio. | Please refer to line 362 for the thickness of the containment as 0.9 m. The equivalent diameters for horizontal and vertical steel bars are 63.2 mm and 58.3 mm, respectively. The total reinforcement ratio is 2.4 %. The said highlighted text is included in lines 173-175 of revised manuscript.  In actual containment, there are three layers of both horizontal/circumferential and vertical bars with 32 mm and 40 mm diameters. However, in current study the two layers (outer and inner) are modeled with equivalent diameters mentioned above to reduce the element count and, therefore, keep the computational time manageable, while preserving the overall reinforcement ratio as per the actual case. |
| 7. | How to set the bottom constraint of containment when modeling the finite element model? | While modelling, the bottom nodes of all elements (concrete and rebars) are constrained for both translations and rotations degree of freedom to achieve fixed boundary conditions. The said highlighted text is included in lines 177-179 of revised manuscript, please. |
| 8. | When the force time history is loaded on the containment, the mesh size of the containment is subdivided. How is it divided and what is the mesh size? | It appears that the related text might have some confusions. Therefore, lines 355-357 of manuscript are rephrased for better clarity. The selected mesh size of containment is 0.6 m as mentioned in line 172 of manuscript and the same is adopted in loading area/impact area as shown in Fig.1 to response 5. |
| 9. | How are keywords of *MAT_NULL and *EOS_GRUNEISEN set in LS-DYNA for this  Paper ? | For current study, in LS-DYNA, the input parameters shown in Table 1 for *MAT_NULL and *EOS_GRUNEISEN are set considering the recommendations given in LS-DYNA keyword manual [5] and review of similar published studies, e.g. [10, 11]. The above highlighted text is added in section 2.1 (lines 159-162, page 7) and input parameters/keywords are reflected in Table 1 (page 23) of revised manuscript, as suggested please. It is also reiterated that the defined aircraft model was verified for its accuracy against impact on rigid wall and results were compared with analytical Riera equation. This may further verify the suitability of input parameters/keyword for said material model, i.e. *MAT_NULL, please. |
| 10. | When comparing impact displacement, concrete stress and reinforcement stress obtained with two methods, please explain the respective time of occurrence. Is there a big difference between two time of occurrence? Why? | The comparison of two methods is made in Figs. 25-29 of section 6. Due to involved dynamics and complex nature of problem along-with difference in the two methods, the results keep changing over time. For results evaluation, very careful inspection in LS-PREPOST (post-processor of LS-DYNA) was performed in order to get maximum possible information from simulation analysis. The critical parameters were compared based on different factors. For example, in Fig. 26, both stresses are taken corresponding to maximum impact forces mentioned in Fig. 11 (a), which is almost at same time of occurrence. Likewise, comparison of damaged elements in Fig. 27 and axial stresses for reinforcements in Fig. 28 are made at end of analysis to have the worst case scenario. Accordingly, the captions of Figs. 25-29 in the revised submission as suggested, please. |
| 11. | In Fig.12 and Fig.15, are they resultant velocities of damaged parts? I think they may be residual velocities of uncrushed parts of aircraft. | The authors agree with the respected reviewer. Yes, these are the residual velocities of uncrushed aircraft parts. The same is also defined in line 213 of manuscript, i.e. $\upsilon(t)$ *is the velocity of the aircraft which has not been crushed*. Accordingly, the necessary corrections are made in text and related Figs. 12 and 15 (now these are Figs.14 and 17 in revised manuscript) as shown in track change mode, please. Both in text and said figures, the term resultant and damaged are replaced with residual and uncrushed, as highlighted please. |
| 12. | In Fig.16 and Fig.20, is the selected node on steel or concrete? The displacement of scattered debris from impact area will be great without any obstruct, because the scattered debris may get an initial velocity in the impact process. It is better to give the maximum displacement of the impact area instead of scattered debris. | The authors do agree with the respected reviewer. The selected node is on concrete in the impact area, and not from scattered debris. The relevant text and caption of Fig. 18 (previously Fig. 16) are modified as suggested, please. In Fig. 22 (previously Fig. 20), the circle indicates the displacement time history and contours of maximum displacement at the impact area, please. |

**References**

1.	Riera, J.D., *On the stress analysis of structures subjected to aircraft impact forces.* *Nuclear Engineering and Design* 1968. **8**: p. 415-426.

2.	Abbas, H., et al., *Reaction-time response of aircraft crash.* Computers & Structures, 1995. **55**(5): p. 809-817.

3.	Zhang, T., et al., *Influences of nuclear containment radius on the aircraft impact force based on the Riera function.* Nuclear Engineering and Design, 2015. **293**: p. 196-204.

4.	Liu, J. and P. Han, *Numerical Analyses of a Shield Building Subjected to a Large Commercial Aircraft Impact.* Shock and Vibration, 2018. **2018**: p. 1-17.

5.	LSTC, *Livemore Software Technology Corporation (LSTC), LS-DYNA Keyword User’s Manual*. 2010.

6.	ERIN Engineering & Research Inc., *Methodology for performing aircraft impact assessments for new plant designs*, in *NEI 07-13, Revision 8P*. 2011: 2001 N. Main Street, Suite 510, Walnut Creek, CA 94596. p. 69.

7.	Mizuno, J., et al., *Investigation on impact resistance of steel plate reinforced concrete barriers against aircraft impact Part 1: Test program and results*, in *18th International Conference on Structural Mechanics in Reactor Technology (SMiRT 18-J05-1), Beijing, China.* 2005.

8.	Sadiq, M., Z.X. Yun, and P. Rong, *Simulation analysis of impact tests of steel plate reinforced concrete and reinforced concrete slabs against aircraft impact and its validation with experimental results.* Nuclear Engineering and Design, 2014. **273**: p. 653-667.

9.	Arros, J. and N. Doumbalski, *Analysis of aircraft impact to concrete structures.* Nuclear Engineering and Design, 2007. **237**(12-13): p. 1241-1249.

10.	Wilt, T., A. Chowdhury, and P. Cox, *Response of reinforced concrete structures to aircraft crash impact.* Prepared for US Nuclear Regulatory Commission Contract NRC-02-07-006, 2011.

11.	Wierzbicki, T., L. Xue, and M. Hendry-Brogan, *Aircraft Impact Damage*, in *The Towers Lost and Beyond, A collection of essays on the WTC by researchers at the Massachusetts Institute of Technology*. 2002. p. 31-64.

Annexure-I
